# Computational Approaches to Cancer Cell Dormancy: From Detection to Dynamic Modelling

**DOI:** 10.3390/biom16050633

**Published:** 2026-04-24

**Authors:** Lucas G. N. Spink, Shi Pan, Minyoung Kim, Belis Yener, Borbála Bánfalvi, Maria Secrier

**Affiliations:** UCL Genetics Institute, Department of Genetics, Evolution and Environment, University College London, Gower Street, London WC1E 6BT, UK; lucas.spink.22@ucl.ac.uk (L.G.N.S.); shi.pan@ucl.ac.uk (S.P.); minyoung.kim.23@ucl.ac.uk (M.K.); belis.yener.23@ucl.ac.uk (B.Y.); b.banfalvi@qmul.ac.uk (B.B.)

**Keywords:** cancer cell dormancy, computational modelling, cell state transitions, tumour reactivation, drug-tolerant persister cells, disseminated tumour cells

## Abstract

Cancer cell dormancy is a clinically consequential yet computationally under-defined phenomenon characterised by reversible growth arrest and delayed disease recurrence. Although advances in single-cell and multi-omic profiling have improved detection of dormant and persister populations, their molecular identity and dynamical behaviour remain difficult to resolve. In this review, we examine how computational methods have been applied to infer dormant cell identity, heterogeneity, microenvironmental regulation, state transitions, and reactivation dynamics. We highlight how single-cell transcriptomics, lineage tracing, spatial profiling, and integrative multi-omic analyses reveal substantial context-dependent variability, undermining the notion of a universal dormancy signature. We further discuss emerging mathematical and statistical frameworks to model the awakening from dormancy, alongside approaches linking dormancy-associated features to clinical outcomes. Recurring challenges include fragmented operational definitions, rare-state detection, cross-study incompatibility, and the use of snapshot data to interrogate inherently temporal processes. We argue that progress will depend on computational frameworks that treat dormancy as a dynamic, multi-scale systems problem rather than a static cell-type classification task.

## 1. Introduction

Cellular dormancy is an evolutionarily ancient survival strategy. Across many domains of life, cells have evolved the capacity to enter reversible growth arrest in response to environmental adversity, enabling them to endure otherwise lethal conditions and resume proliferation when favourable conditions return [1,2]. In yeast, nutrient limitation triggers entry into the stationary phase, a quiescent state regulated by conserved nutrient-sensing pathways, including TOR, PKA, and AMPK [3,4,5,6]. In the nematode *Caenorhabditis elegans*, larvae enter the stress-resistant dauer larval state under unfavourable conditions, a process regulated by insulin/IGF-1 signalling (IIS) and AMPK pathways, which converge on the transcription factor DAF-16, as well as the PKA pathway [7,8,9]. Some killifish species live in ponds that desiccate during dry seasons; to survive these periods, killifish embryos undergo diapause, a prolonged developmental arrest that is thought to be mediated by the IIS, AMPK, and p53 pathways, enabling survival through seasonal desiccation [10,11,12,13,14]. In mammals, quiescence is regulated by the p53 and mTOR pathways, integrated with the IIS and AMPK signalling networks [15,16,17,18]. These pathways share not only functional logic but also specific molecular components: the AMPK pathway is significant in quiescence regulation across all multicellular eukaryotes examined, while TOR, IIS, and p53 are conserved across overlapping but distinct subsets of species. Similarly, the phenotypic hallmarks of quiescent cells, including arrested growth, decreased cell size, reduced biosynthesis, increased autophagy, and resistance to environmental stresses, are remarkably consistent across organisms [14,19].

This evolutionary conservation is directly relevant to cancer biology. Rather than representing a cancer-specific process, cellular cancer dormancy (not to be confused with population-level forms of dormancy, such as angiogenic or immunogenic dormancy [20]) is best understood as the pathological co-option of these conserved cellular stress-response and developmental quiescence programmes [2]. Cancer cells that enter dormancy exploit pre-existing survival mechanisms that normally enable cells to withstand environmental stress, nutrient limitation, or unfavourable growth conditions [21,22,23]. In doing so, they become refractory to therapies targeting actively proliferating cells and can persist undetected for extended periods before potentially reawakening to seed disease recurrence, often years or decades after apparently successful treatment [24,25,26]. Understanding cancer dormancy is therefore of considerable clinical importance, as dormant cancer cells represent a reservoir for relapse and metastasis [27], yet they remain poorly characterised and largely invisible to current therapeutic and diagnostic strategies.

**Figure 1:**
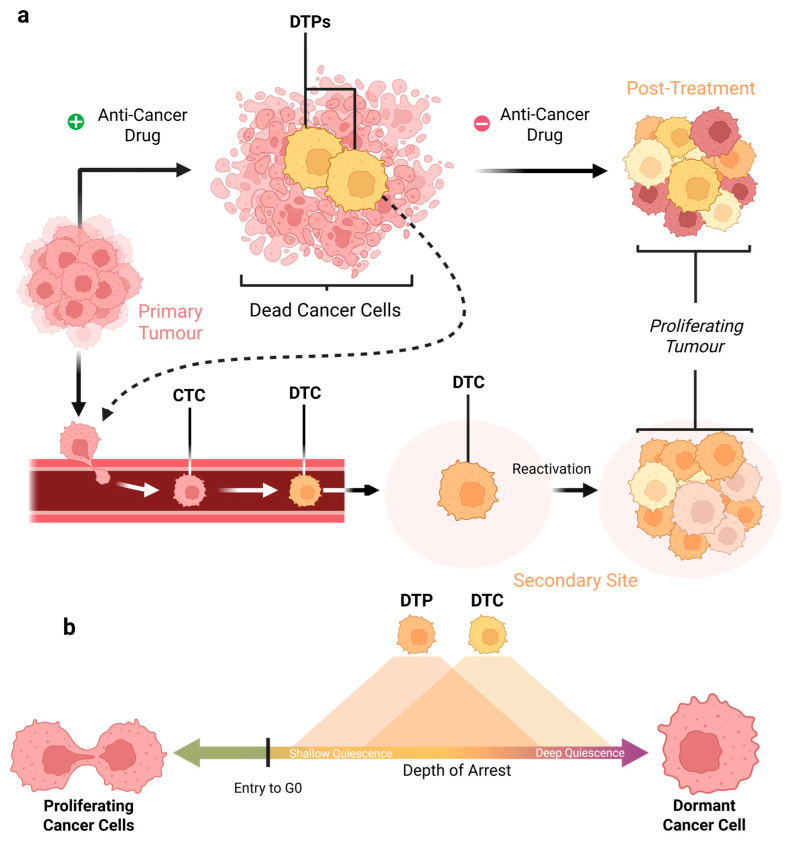
Mechanisms of drug-tolerant persister (DTP) and disseminated tumour cell (DTC) formation, and a proposed spectrum of dormant cancer cell states across varying depths of quiescence. (**a**) Schematic representation of the origins and fates of two different manifestations of cancer dormancy, DTPs and DTCs. DTPs form from anti-cancer treatment that pushes the cancer cells into a dormant state that allows them to survive therapy. After treatment is removed, DTPs re-enter the cell cycle and begin to proliferate again, forming new tumours. DTCs are circulating tumour cells (CTCs) that have become dormant and then embedded in a secondary site, where they remain dormant for a period before beginning to proliferate and form a metastatic tumour. It is important to note that DTPs can also leave the primary tumour to form metastases in a similar fashion. Arrows indicate transitions between proliferative and dormant states. (**b**) Conceptual model of a dormancy continuum, illustrating a spectrum of cell states with increasing depth of quiescence and significant overlap between dormant subtypes, rather than discrete categories. Created in BioRender. Spink, L. (2026). https://BioRender.com/f05zqdp (accessed on 17 April 2026).

In cancer, cellular dormancy refers to treatment-refractory cancer cells that have entered reversible G0/G1 arrest, with varying levels of metabolic activity [25,27,28,29,30,31]. While the general characteristics of dormant cancer cells are reasonably well established in the literature, the terminology used to describe them remains inconsistent and often overlapping, making precise discussion challenging [32]. Two manifestations of cancer dormancy have been widely characterised in the literature (Figure 1a):

**Drug-Tolerant Persisters (DTPs)** describe a subpopulation of cancer cells that survive cancer treatment that would otherwise be lethal through non-genetic mechanisms. Instead of acquiring resistance mutations, DTPs enter reversible G0 or slowly cycling (long G1) states, enabling them to withstand the drug challenge via epigenetic changes or metabolic rewiring. These cells are thought to form a reservoir of resistance, as they can revert to proliferation after selective pressure from cancer treatment is removed (Figure 1a) [33,34].

**Disseminated Tumour Cells (DTCs)** are cancer cells that enter the blood as circulating tumour cells (CTCs) and then embed themselves in organs and tissues [35]. These DTCs will typically have entered reversible G0/G1 arrest and, once embedded, will remain clinically silent for long periods before “reawakening” to form metastases (Figure 1a). Similar to DTPs, DTCs are also thought to be a key driver of cancer recurrence [36].

Alongside these, evidence of other forms of dormancy in the context of therapeutic resistance is beginning to emerge in multiple cancer types:

**Polyploid Giant Cancer Cells (PGCC)** are a subpopulation of cancer cells characterised by giant or multiple nuclei and polyploidy [37]. PGCCs are thought to result from endoreduplication, cell fusion, cytokinesis failure, and mitotic slippage induced by various stimuli, including therapeutic drugs, radiation treatment, hypoxic environments, and human oncoviruses and cytomegaloviruses. Recent work has also linked their emergence to so-called “treacherous apoptosis”, in which cells initiate but fail to complete apoptotic programmes, instead surviving and contributing to tumour heterogeneity and repopulation, suggesting that cell death pathways themselves may paradoxically promote the formation of therapy-resistant, dormant-like states [38,39]. When environmental conditions are unfavourable, PGCCs enter a slow-cycling/quiescent state, becoming drug-resistant. PGCCs were long considered terminally senescent and incapable of further division; however, pioneering work by Walen and Rajaraman and colleagues in the early 2000s demonstrated that polyploid cells can undergo amitotic division to produce viable, rapidly proliferating progeny with tumorigenic potential [40]. When conditions are favourable again, PGCCs exit their dormant state. They can begin to produce progeny via asymmetric non-canonical divisions (e.g., budding, splitting, bursting, neosis, and amitosis), a process dubbed depolyploidisation. The resulting daughter cells exhibit cancer stem-like properties, high invasion/migration, and chemoradiotherapy resistance and are capable of initiating tumours in vivo [37,41,42,43]. Moreover, PGCC-derived daughter cells have been repeatedly identified as major drivers of tumour recurrence and distant metastasis [44,45,46,47].

**Stably Resistant Cancer Cells (SRCCs)** are a *NOTCH*+ subpopulation of dormant cancer cells described in lung cancer exhibiting stable, innate resistance to chemotherapy, targeted therapy, and immunotherapy. SRCCs’ cellular and molecular characteristics, such as epithelial state, elevated reactive oxygen species, and anti-apoptotic and anti-ferroptotic survival, differentiate them from other resistant populations, such as cancer stem cells, epithelial–mesenchymal transition cells, and DTPs. Furthermore, inhibition of the non-canonical *NOTCH1* pathway resensitises these cells to cisplatin-induced cell death in vitro and in vivo [48].

While several forms of cancer cell dormancy have been proposed, these definitions overlap. For example, a DTC that has been exposed to therapy could simultaneously meet the definition of a DTP and DTC. Similarly, a DTP exposed to sufficiently high levels of environmental stress could take on characteristics associated with PGCCs. These overlaps have prompted some to ask whether these subpopulations truly represent biologically distinct cellular types or whether cancer dormancy may be better represented by a spectrum of states along a gradient of cell cycle arrest depth (Figure 1b) [29]. While we lack exact and discrete definitions for the different forms of dormancy in cancer, several biological programmes have been linked to the entry to, maintenance of, and exit from this state.

### Known Mechanisms of Cancer Dormancy and the Need for Computational Approaches

The molecular mechanisms underlying cancer dormancy can be broadly organised into three functional categories: induction of dormancy, maintenance of this state, and reawakening into proliferation. Induction of dormancy is thought to be triggered by a range of factors, including intrinsic cellular stress and microenvironmental cues, and is enacted through cell cycle arrest, TGF-β/BMP signalling, and downregulation of oncogenic pathways [24,49,50,51,52]. A well-established hallmark of dormancy is the balance between ERK and p38 signalling. Loss of integrin-mediated extracellular matrix signalling, hypoxia, nutrient stress, and cancer therapy are thought to promote a shift from high ERK/low p38 activity towards low ERK/high p38 activity, associated with entry into quiescence, a transition regulated in part by upstream mediators such as uPAR [51,52,53,54,55,56].

Maintenance of dormancy is primarily thought to be mediated by continued p27/p21 expression, low CDK activity, and sustained low ERK/high p38 signalling, which keep cells in G0 [30,52,54]. Autophagy, metabolic changes, and reduced biosynthesis are also thought to be modified to support long-term survival with minimal accumulation of damage [51,57]. In parallel, stress-response pathways such as the unfolded protein response contribute to dormancy maintenance, in part through activation of the antioxidant regulator NRF2, which mitigates oxidative stress associated with altered metabolism and has been implicated in defining distinct persister subpopulations [58,59]. At the transcriptional level, factors such as NR2F1, Dec2, and NDRG1 further reinforce this state by coordinating gene programmes associated with quiescence, stemness, and stress adaptation [60,61,62,63]. The niche in which the dormant cancer cells reside is also thought to be important to maintenance, with evidence that specific extracellular matrix (ECM) composition, integrin signalling, and perivascular/bone-marrow niches actively help maintain quiescence [24,64]. These pathways form an integrated network rather than independent modules, collectively stabilising reversible growth arrest while preserving long-term cellular viability.

The mechanisms surrounding dormant cancer cells reawakening are more nebulous, but reawakening is thought to be partly mediated by the reversal of the aforementioned programmes (e.g., restoration of the ERK/p38 balance); however, exact triggers for reawakening remain unclear [30]. One example of a potential pro-reawakening trigger is ECM remodelling and chronic inflammation, which provide pro-proliferative cues that could potentially trigger re-entry to the cell cycle, restarting tumour proliferation [65].

While previous experimental work in the field has revealed some pathways and general principles of cancer dormancy, as comprehensively reviewed elsewhere [27,49,66,67,68], these findings have yet to converge on a unified mechanistic framework, and much of this process remains poorly understood. Recent advances in single-cell and multi-omic profiling technologies, and the computational techniques to analyse their output, have shown promise in helping us better understand cancer dormancy. In this review, we begin by discussing a series of key questions surrounding cancer dormancy and how computational techniques have been used to address them, particularly by enabling the identification, stratification, and dynamic modelling of rare dormant cell populations that are otherwise inaccessible to bulk experimental approaches. We then go on to discuss the main challenges for computational analysis of cancer dormancy as well as potential future solutions to these challenges. Given the overlapping definitions and lack of consensus within the field, throughout this review, we use the term “dormant cancer cells” to broadly refer to cancer cells that have, through any mechanism, entered reversible G0/G1 arrest, rendering them refractory to standard cancer treatment. Where a study focuses on a specific subtype, we use the specific term (e.g., DTC, DTP, etc.). This usage is not intended to imply that these subtypes are biologically equivalent but reflects the current state of the field in which the precise differences between the dormant cancer cell subtypes remain unresolved.

## 2. From Multi-Omics Data to Mathematical Models: Computational Studies of Dormancy

**Figure 2:**
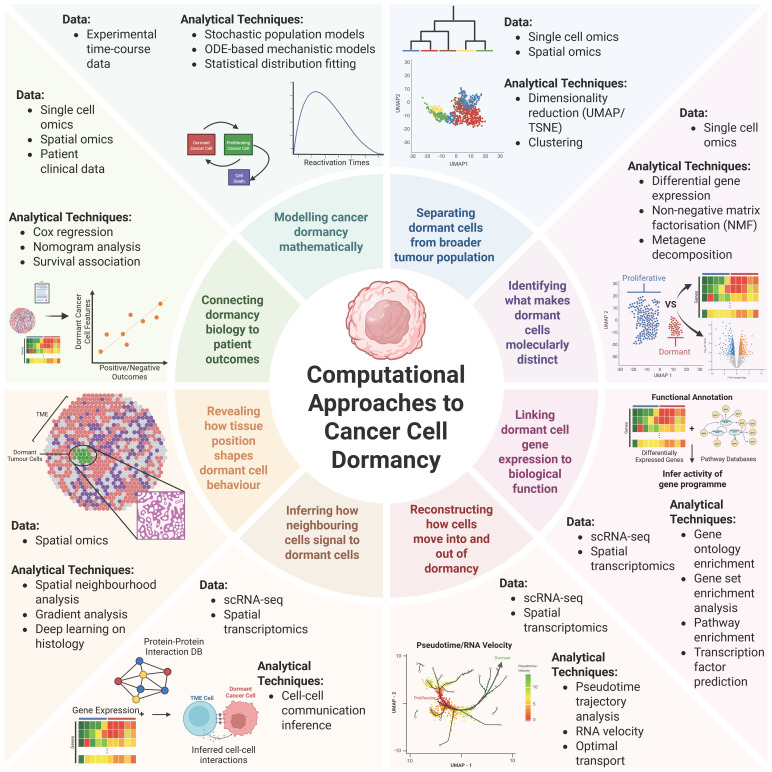
Overview of computational approaches to studying cancer cell dormancy. Eight key analytical strategies for interrogating cancer cell dormancy are arranged around a central theme, each accompanied by a schematic illustration, the data modalities it draws on, and the specific techniques it encompasses. Starting from the top and moving clockwise: (1) dimensionality reduction and clustering of single cell or spatial omics data separates dormant cells from the broader tumour population and identifies subtypes within them; (2) differential gene expression analysis, transcription factor prediction, and metagene decomposition across single cell omic data identify what makes dormant cells molecularly distinct; (3) gene ontology, gene set enrichment, and pathway enrichment analyses link dormant cell gene expression changes to biological function; (4) pseudotime trajectory analysis and RNA velocity applied to single-cell and spatial transcriptomic data reconstruct how cells transition into and out of dormancy; (5) cell–cell communication inference from single-cell expression data, combined with protein–protein interaction databases, infers how neighbouring cells in the tumour microenvironment signal to dormant cells; (6) spatial neighbourhood analysis, gradient analysis, and deep learning on histopathology applied to spatial transcriptomic data reveal how tissue position shapes dormant cell behaviour; (7) Cox regression, nomogram analysis, and survival association applied to single-cell/spatial omic and clinical data connect dormancy biology to patient outcomes; (8) stochastic population models, ODE-based mechanistic models, and statistical distribution fitting applied to experimental time-course data model cancer dormancy dynamics mathematically, including reactivation timing. No single data type or analytical method is sufficient to capture the complexity of cancer cell dormancy, and progress in the field relies on the complementary application of multiple approaches across different data modalities. Created in BioRender. Spink, L. (2026) https://BioRender.com/00foaaz (accessed on 17 April 2026).

Dormant cancer cells are widely considered to be rare in tumour populations [50]. As such, in bulk sequencing datasets, dormant cancer cells are averaged out and are essentially invisible. Advances in single-cell resolution and spatially resolved technologies, including single-cell RNA sequencing, single-cell multi-omics, and spatial transcriptomics, have enabled the detection and characterisation of dormant cancer cells within heterogeneous tumour populations. In doing so, these technologies also revealed the extent of the challenge that dormant cancer cells represent: they are rare in most datasets, difficult to distinguish from other cell cycle arrest states (e.g., quiescence, senescence, etc.), and their regulatory programmes are context-dependent. In the following sections, we examine how computational approaches have been applied to four central questions in cancer dormancy research: inference of dormant cancer cell identity, microenvironmental regulation of dormancy, transitions into and out of the dormant state, and the influence of cancer dormancy on clinical outcomes (Figure 2).

### 2.1. Computational Inference of Dormant Cell Identity

One of the first major challenges in computational research on cancer dormancy is reliably identifying dormant cancer cells. All computational studies of dormancy must first define which cells are dormant, and in the absence of a consensus molecular marker, this definition varies across studies. Some studies use label retention as a proxy for quiescence, while others use survival under sustained drug treatment or growth arrest under therapy. Given this definitional challenge, many computational studies have attempted to identify markers for dormant cancer cells. Here, we discuss a series of papers that apply computational techniques across different modalities to characterise dormant cancer cells.

A natural starting point is to ask whether dormant cancer cells express distinct genes that could serve as molecular markers. To answer this question, Ren et al. [69] used the retention of the membrane dye DiD as a functional readout of proliferative history (quiescent cells retain the membrane dye, while actively dividing cells dilute it) to distinguish proliferative from dormant metastatic breast cancer cells in the lungs and bone marrow of a genetically engineered mouse model. The proliferative and dormant cells were then sequenced using single-cell RNA sequencing (scRNA-seq), and differential gene expression was performed, identifying a group of genes, including *Cfh*, *Gas6*, *Mme*, and *Ogn*, that were all upregulated in dormant breast cancer cells compared to the proliferative breast cancer cells. While these genes correlated with disease-free survival in breast cancer patients, attempts to validate these results experimentally with single-gene perturbation experiments in murine models showed that no individual gene affected dormancy, indicating that dormancy is likely not controlled by a single, universal master regulator but reflects a coordinated programme-level change which can be difficult to capture through standard differential analysis, particularly if some of the changes are short-lived. Moreover, the genes identified by Ren et al. are likely specific to metastatic breast cancer in this specific experimental context. Indeed, across the broader literature, the wide range of dormancy-associated markers that have been reported reinforces the view that many dormancy programmes are context-dependent, even if certain core gene programmes are shared.

While marker-based isolation followed by transcriptomic profiling identifies candidate genes, combining lineage tracing with multi-modal profiling can reveal additional molecular layers of dormant cell identity that transcriptomics alone cannot capture. For example, Oren et al. [70] developed Watermelon, a lentiviral barcoding system that combines lineage tracing with scRNA-seq, and applied it to lung, breast, melanoma and colorectal cancer cell lines treated with clinically relevant kinase inhibitors (osimertinib, lapatinib or dabrafenib) to track the emergence of persistent cells. These DTPs were separated into two phenotypes, cycling and non-cycling persisters, which arose from distinct cell lineages. Subsequent differential expression analysis of scRNA-sequencing and LC-MS/MS metabolic profiling showed that the rare subset of cells capable of re-entering the cell cycle under constitutive drug treatment (cycling persisters) presented nuclear factor erythroid 2-related factor 2 (*NRF2*)-driven antioxidant programmes and a shift towards fatty acid oxidation, which were absent in the non-cycling persisters. The authors demonstrated that these signatures are partially or fully reproducible across multiple lines and in patient tumour samples, indicating that cycling persisters share a reproducible molecular identity defined by antioxidant and metabolic reprogramming and identifying these programmes as potential markers to distinguish cycling from non-cycling persister populations.

While Oren et al.’s combination of transcriptomic and metabolomic profiling with lineage tracing identified *NRF2*-driven antioxidant programmes and metabolic reprogramming as defining features of cycling persisters, these signatures only operate at the transcriptomic and metabolomic levels. Rosano et al. [71] extended this multimodal approach to the epigenetic layer. In their TRADITIOM (**TR**acking Adaptation, **D**ormancy and Awaken**i**ng with mul**tiom**ics) study, the authors developed a long-term in vitro lineage-tracing system that incorporates bulk RNA-seq and scRNA-seq, whole-genome sequencing, and super-SILAC (stable isotope labelling by amino acids in cell culture) histone mass spectrometry [72] to map the adaptive trajectories of ER^+^ breast cancer cell lines into and out of dormancy under long-term endocrine therapies. By combining clonal barcode tracking with transcriptomic profiling over extended treatment periods, this work demonstrated that endocrine therapies can induce a non-genetic transition into dormancy in a stochastic subset of cells and that individual dormant lineages can later reawaken through divergent epigenetic reprogramming without recurrent genetic mutations. This approach revealed that dormant cell identity in this system operates primarily at the chromatin level and that epigenetic profiling captures aspects of dormancy invisible to transcriptomics or genomics alone. Together, Oren et al. and Rosano et al. illustrate that dormant cancer cell identity spans multiple molecular layers (transcriptomic, metabolic, epigenetic) and that no single modality can capture the full picture. They also illustrate a key tension in the field: whether dormant cell identity is defined by stable, pre-existing molecular programmes, as Oren et al.’s reproducible *NRF2* signatures suggest, or by variable epigenetic states that differ across individual dormant lineages, as Rosano et al. found. These conclusions are not incompatible but rather reflect differences in cancer type and treatment regimen; they have significant implications for whether dormancy can be predicted from the pre-treatment state or only observed retrospectively.

Taken together, these studies show that while computational approaches can identify and stratify dormant and persister populations at single-cell resolution, they do not converge on a stable or universal dormancy signature. Instead, they reveal substantial lineage- and context-specific heterogeneity, with different molecular programmes supporting similar phenotypic outcomes. This reinforces the view that dormancy may not represent a single, fixed cell state, but rather a flexible, adaptive state whose identity is contingent on prior history and environmental conditions.

### 2.2. Computational Analysis of Microenvironmental and Spatial Regulation of Cancer Dormancy

Another key question in the field is how the microenvironment and spatial context may influence cancer dormancy. The idea that the microenvironment was involved in cancer was first put forward by Steven Paget with his “seed and soil” hypothesis in 1889, where he suggested that the spread of tumour cells across the body to form metastases was mediated by an interaction between cancer cells (seed) and the organs/tissues they embedded in (soil) [73]. Paget’s hypothesis has proved especially relevant to dormancy, where niche-derived signals have been implicated in both the induction and the disruption of quiescent states. Recent advances in single-cell and spatial profiling technologies, combined with increasingly sophisticated computational approaches, have enabled researchers to interrogate these interactions at high resolution. Here, we discuss a series of studies that have applied such approaches to characterise the microenvironmental regulation of dormancy at increasing scales of complexity, from individual niche interactions to pan-cancer network analyses.

Initial computational studies examining how the microenvironment may regulate cancer dormancy primarily utilised scRNA-seq data alongside various computational analyses. While these studies consistently found that the tumour microenvironment (TME) plays a role in cancer cell dormancy, the manner in which the TME affects cancer cell dormancy differed. For example, Khoo et al. [74] sought to understand how the endosteal bone microenvironment affected myeloma cell dormancy. They experimentally identified dormant and activated myeloma cells in murine bone marrow using DiD label retention as a readout of proliferation and then sequenced them using scRNA-seq. The authors then analysed the scRNA-seq data with differential gene expression analysis, gene ontology enrichment, and transcription factor prediction. They also applied non-negative matrix factorisation (NMF) clustering, which decomposes gene expression data into a small number of interpretable gene expression patterns and shows how strongly each pattern is present in each sample [75]. Their analysis showed that, despite myeloma cells being of lymphoid origin, the dormant myeloma cells had a transcriptional profile enriched for immune-related genes and myeloid differentiation genes. Experimental validation using coculture and intravital imaging showed that the myeloid transcriptional reprogramming was induced by direct contact with osteoblasts. Inhibition of *AXL*, one of the highly expressed genes found in dormant myeloma cells, reduced dormant cancer cells in their mouse model. In patients with monoclonal gammopathy of uncertain significance, enrichment of *AXL* and genes coregulated with *AXL* expression was associated with better long-term survival compared to controls, consistent with a greater proportion of cancer cells being in a quiescent state. Furthermore, the expression of the myeloid transcriptional signature in patients with multiple myeloma was associated with a twofold increase in overall survival. This study showed that the environment/niche can significantly affect dormant cancer cells and that computational tools can effectively model these interactions.

While Khoo et al. identified specific cell–cell interactions driving cancer cell dormancy-associated reprogramming, Janghorban et al. [76] found evidence of broader TME remodelling. Janghorban et al. generated GFP+ tumours in GFP mice, which were then transplanted into the fat pads of immunogenic mice. After the tumours reached 7–9 mm in diameter, the mice were treated with chemotherapy (FGFR inhibitor) for 14 days. Mice were sacrificed, and their tumours and surrounding microenvironments were sequenced with scRNA-seq at 3 time points: before treatment, after 14 days of chemotherapy treatment and 14 days off treatment, and 1–4 months after treatment, representing primary, dormant, and recurrent populations of cancer cells. Some mice were also treated with α-Jag1-blocking antibodies or with α-IgG2b. The authors then performed differential gene expression, trajectory analysis, and gene ontology analyses, which showed that cancer dormancy was associated with significant remodelling of the myeloid and lymphoid compartments. Among newly recruited myeloid and lymphoid cells during dormancy, the majority exhibited immunosuppressive activity. They further showed that Jagged-1/Notch signalling played a key role in dormancy, as treatment with anti-Jag1 antibodies delayed tumour recurrence. Ultimately, this study demonstrated that dormancy was associated with broader changes in TME composition compared to the specific interactions described in Khoo et al. (albeit in a different system).

These studies raise an important question about the scale at which the TME regulates dormancy. In Khoo et al.’s myeloma study, reprogramming effected by the TME appears to be driven by a specific cell–cell interaction between osteoblasts and dormant cancer cells in the bone marrow niche. In Janghorban et al.’s mouse breast cancer model of minimal residual disease, dormancy is associated with broader remodelling of the entire TME. Whether this difference in interactions truly represents a distinct regulatory mechanism or reflects differences in tissue context between bone marrow and mouse mammary fat pad remains unclear.

While the previous two studies used scRNA-seq and standard bioinformatics analyses to investigate how the microenvironment contributes to cancer dormancy, scRNA-seq is blind to the spatial context of these microenvironments. Recent advances in spatial transcriptomics now allow researchers to explore both the transcriptomic and spatial context of dormancy. Rubinstein et al. [77] utilised spatial transcriptomics to explore how the spatial organisation of the tumour microenvironment affects drug-induced dormancy in BRAF-mutant melanoma. The authors used patient-derived xenograft models treated with dabrafenib/trametinib to simulate treatment with samples collected at various time points. Computationally, the authors applied copy number alteration inference and pathway enrichment to profile the molecular characteristics of the dormant cells. They also used pseudotime trajectory analysis, which orders cells along an inferred temporal axis based on transcriptomic similarity [78], and RNA velocity, which estimates future cell states by comparing spliced and unspliced RNA ratios [79], to reconstruct cell state transitions. Their results showed that after treatment dormant cancer cells were characterised by increased oxidative phosphorylation, decreased proliferation, and increased invasive capacity. Strikingly, the authors also found central-peripheral patterns in tumours, where populations at the tumour-stromal boundary were subject to distinct signalling from those in the intra-tumoural regions and, as a result, exhibited distinct transcriptional changes. Crucially, these central-to-peripheral gradients would have been invisible in dissociated scRNA-seq experiments, as tissue dissociation removes the spatial context. Furthermore, deep learning applied to histopathology slides demonstrated that major transcriptional states, treatment-sensitive versus resistant lineages, and the persister phenotype could be distinguished directly from H&E images, despite being indistinguishable by conventional pathological review, with imaging features tracking disease progression through treatment and correlating strongly with RNA expression clusters and, more weakly, with CNV-defined lineages. This raises the possibility that dormancy-associated spatial patterns may eventually be detectable from standard clinical imaging.

Beyond spatial organisation, an increasingly common complementary approach in single-cell and spatial transcriptomics is ligand–receptor-based inference of cell–cell communication (Figure 2). Although widely used to characterise tumour microenvironment signalling, its direct application to cancer dormancy remains limited. Given the central role of niche-derived signalling, this represents a promising direction for identifying microenvironmental regulators of dormancy, with emerging work in related contexts beginning to explore this space [80].

Together, these studies illustrate how computational approaches at increasing molecular resolution have reshaped our understanding of the microenvironmental regulation of dormancy. From niche-specific transcriptional reprogramming in myeloma to immune ecosystem remodelling in breast cancer to spatially resolved metabolic gradients in melanoma, a consistent theme emerges: the microenvironment does not merely provide a passive sanctuary for dormant cells but actively shapes their transcriptional identity, immune interactions, and metabolic state.

### 2.3. Computational Inference of Dormancy State Transitions

Beyond identifying cell states and interactions with the microenvironment, another poorly understood aspect of cancer cell dormancy is the mechanism(s) underpinning entry into and exit from cancer cell dormancy. Specifically, two distinct questions remain unanswered: what are the molecular mechanisms driving entry, maintenance, and exit from the dormant state, and whether the dormant state itself represents a single uniform condition, a graded spectrum of quiescence depth [81], or a set of biologically distinct subtypes. These are clinically relevant questions, as the factors that govern these transitions are prime targets for treatments aimed at reducing the risk of relapse and metastasis. As these transitions are difficult to observe directly in patients and unfold on time scales that are difficult to capture experimentally, computational inference from high-resolution molecular data offers one of the few available routes to study them effectively. Despite its clinical importance, studies directly investigating how this transition occurs computationally remain limited; however, some work has begun to move in this direction.

Fernandez et al. [82] combined scRNA-seq and time-lapse imaging of cell cycle dynamics to examine the range of quiescent/senescent phenotypes in MCF10A mammary epithelial cells following chemotherapy (etoposide). Cells were exposed to increasing doses of etoposide, and *CDK2* activity was used to classify fast-cycling, slow-cycling (quiescent), and arrested (senescent) cells, which were then profiled by scRNA-seq on day 6 after drug washout. The authors then used a combination of dimensionality reduction, clustering, differential gene expression, and pseudotime trajectory analysis to analyse the scRNA-seq data. Strikingly, instead of forming transcriptionally distinct islands, the pseudotime analysis applied by the authors showed that etoposide-treated cells formed a continuous trajectory of cells that gradually tended towards deeper arrest. Gene set enrichment analysis (GSEA) showed progressive downregulation of cell-cycle, RNA-processing, and protein-translation programmes along the pseudotime axes, supporting the idea that cells treated with therapy enter arrest gradually rather than in a switch-like fashion. From this continuum, the study identified four transcriptionally distinct senescent subtypes, termed “senotypes”, arising from two cellular arrest routes: cells that arrested gradually via a standard mitosis-to-G0 transition and cells that arrested via mitotic slip. While this study was performed in non-cancerous cell lines, these findings have implications for drug tolerance, persistence after treatment, and, indirectly, for cancer dormancy. Namely, that rather than drug-induced adaptation representing a binary transition into a uniform dormant state, drug-induced arrest may represent a graded progression from a shallow quiescent state to a deeper, more senescence-like arrest and that these states may represent overlapping rather than discrete categories.

Understanding the mechanisms that regulate the re-entry of dormant cancer cells into the cell cycle is critical for developing treatments that aim to reduce recurrence rates after successful cancer treatment. Hu et al. [83] aimed to elucidate this tumour-intrinsic mechanism using indolent mouse and human lung adenocarcinoma cell lines and patient-derived samples, specifically examining tumour-intrinsic regulators of cancer dormancy. The authors performed in vivo CRISPR–Cas9 screens targeting 220 immune-related genes, combined with sgRNA enrichment sequencing, scRNA-seq, GSEA, chromatin immunoprecipitation sequencing (ChIP–seq), bisulfite sequencing, and functional perturbation experiments. The results implicate the cGAS-STING pathway as an important regulator of dormant cancer cell re-entry to the cell cycle in this model, with STING expression elevated in *SOX2*+ early metastatic progenitors but downregulated in *SOX9*+ macrometastatic cells in both the mouse models and patient-derived data. CHIP-seq showed that STING is epigenetically silenced in metastatic progenitors re-entering the cell cycle via promoter and enhancer hypermethylation, whereas metastatic cells that fail to re-enter the cell cycle and instead re-enter dormancy via TGFβ signalling repress STING through chromatin remodelling. Pharmacological activation of STING reduced dormant metastatic burden in vivo, suggesting that modulation of this pathway may represent a potential strategy to limit lung cancer relapse [83]. Complementing these findings, Rosano et al. [71] further implicates epigenetic reprogramming as a major regulator of dormancy plasticity. As introduced in Section 2.1, they employed long-term, multimodal lineage tracing to track endocrine-treated breast cancer cells as they transitioned in and out of dormancy. The data suggest that entry into dormancy occurs stochastically across lineages through non-genetic mechanisms rather than clonal genetic selection and that reawakening is characterised by divergent epigenetic reprogramming, with different lineages finding distinct chromatin-level routes out of dormancy.

Together, the findings from Rosano et al. and Hu et al. reveal a key unresolved question in the field: when dormant cancer cells reawaken, do they converge on a shared molecular mechanism or awaken via divergent pathways? Hu et al.’s results suggest convergence on epigenetic STING silencing via promoter hypermethylation, consistently associated with reawakening, implying an epigenetic bottleneck to reawakening. Conversely, Rosano et al. support divergent reawakening pathways, with evidence that awakening occurs through distinct epigenetic modifications. This distinction is clinically relevant because, if awakening converges on a single shared mechanism, then, in principle, targeting this mechanism could prevent reawakening across cancers. However, if reawakening is divergent, then no single intervention would be able to prevent reawakening, indicating a need for combination strategies or some form of testing to identify the type of reawakening a specific cancer type utilises. It should be noted that Hu et al.’s and Rosano et al.’s findings come from different cancer types (lung adenocarcinoma and ER+ breast cancer, respectively), different treatments (no treatment and endocrine therapy, respectively), and different experimental time scales. Thus, these divergent results may indicate the context-dependence of cancer cell dormancy reawakening mechanisms.

The studies discussed above showcase how advances in experimental modelling of dormancy, coupled with multi-omics computational analyses, can help elucidate state transitions in cancer dormancy. Beyond standard bioinformatics, cell state transition modelling is an emerging frontier in the field that is powered by increasingly high-throughput, spatially and temporally resolved datasets [81].

### 2.4. Computational Inference of Survival Dynamics and Dormancy Awakening Hazards

Beyond identifying the factors that control entry into and exit from dormancy as targets for therapy, another clinically relevant approach to understanding cancer dormancy is to examine how dormancy affects patient outcomes. Researchers have approached this via two methodologically distinct strategies: forward mathematical modelling and empirical clinical association. Forward mathematical modelling aims to build mechanistic/statistical models from first principles to, for example, predict how dormancy affects treatment efficacy and reactivation timings. In contrast, empirical clinical association studies will work backwards from patient data to see if different features of dormancy correlate with better or worse patient outcomes. Both strategies are ultimately aimed at gaining a better understanding of how to treat patients where cancer dormancy may be causing cancer recurrence more effectively. Here, we discuss how a combination of mathematical modelling and computational analysis can help better understand the influence of cancer dormancy on patient outcomes.

Two studies have recently used mathematical modelling to better understand dormant cancer cell reactivation timings. Blath et al. [84] developed a stochastic individual-based population model to investigate how short-term cancer dormancy affects therapy success by modelling tumour cell populations undergoing therapy. The authors constructed an idealised mathematical model with active and dormant cancer cell compartments and two classes of drug agents, incorporating spontaneous and drug-induced state switching, competition, and drug degradation. Importantly, this model linked phenotypic plasticity (the ability of cancer cells to become dormant) to therapy evasion. Their model showed that even a very small fraction of dormant cancer cells (~0.5%) is enough to prevent tumour eradication under single-drug therapy. They later show that treatment is most effective when targeting dormant cancer cells with a drug that either “wakes” the cell, making it sensitive to traditional chemotherapy, or directly kills the dormant cancer. While the authors acknowledge that this is a toy model that simplifies many aspects of cancer dormancy, it is one of the few examples of mathematical modelling of cellular dormancy to date and among the limited efforts to explicitly incorporate treatment dynamics into the model. As our understanding of the underlying mechanisms of cancer dormancy increases, mathematical modelling will be an increasingly useful tool for understanding how biological knowledge can be translated into effective treatments that overcome dormancy.

Sfakianakis et al. [85] used a dataset from Cameron et al. [86] to investigate which of eight predetermined statistical distributions (Normal, Logistic, Gamma, Weibull, Log-Normal, Pareto, Gompertz, and Inverse Gaussian) best captured the dormancy reactivation dynamics in murine metastatic melanoma. The dataset from Cameron et al. was produced using fluorescently labelled B16F10 murine melanoma cells that were injected into mice and then sacrificed at six time points over 14 days, with the relative frequencies of solitary cells and small, medium, and large cell clusters recorded to characterise dormancy and reactivation dynamics. The authors constructed a mechanistic ODE-based model that included circulating tumour cell death, extravasation, stochastic dormancy duration (sampled from eight distributions), reactivation, proliferation, and tissue-level death, with parameters fitted using particle swarm optimisation. When evaluated against the Cameron et al. dataset, the Pareto and inverse Gaussian distributions provided the best fits, supporting the idea that heavy-tailed statistical representations of dormancy better predict the dynamics of metastatic dormant cell reactivation. However, it is important to note that the model relies on several strong simplifying assumptions: a constant death rate and proliferation rate, an immediate switch from dormancy to full proliferation, and the assumption that cells are independent of one another, which limits biological interpretability. Nevertheless, the study represents a strong step forward towards formalising reactivation dynamics mathematically.

To better understand the consequences of cancer cell dormancy on patient prognosis, some studies have focused on linking measurable characteristics of cancer dormancy (e.g., transcriptomics and the presence of DTCs) to patient outcomes. Tian et al. [87] investigated tumour dormancy in neuroblastoma using a retrospective transcriptomic dataset, performing consensus clustering on a panel of 19 literature-derived dormancy-associated genes to stratify dormant cells into two clusters. The cluster with the higher dormancy signature score showed a significantly lower survival probability over time. The authors then used univariate and multivariate Cox regression analyses to identify dormancy genes associated with prognostic outcomes, resulting in a 6-gene dormancy-associated prognostic signature (*CDKN2A*, *BHLHB3*, *CDKN2B*, *MAPK14*/p38, *CDKN1B*, and *BMP7*) that stratified patients into high- and low-risk groups. Interestingly, several of these genes have been previously implicated in cancer cell dormancy, such as the p38 gene family and *BMP7*, or in cell cycle control (*CDKN2A*, *CDKN2B*, and *CDKN1B*). A nomogram analysis incorporating the six-gene dormancy signature alongside sex, age, stage and N-myc status was used to further split neuroblastoma patients into high-, medium-, and low-risk groups. While this study was mainly correlative and retrospective, it highlights that cancer dormancy biology plays an important role in relapse that can be computationally interrogated to predict patient outcomes.

Tjensvoll et al. [88] used data from an operable non-metastatic breast cancer cohort of 191 patients with a median 15.3-year follow-up to show that the presence of DTCs in bone marrow (BM) before or after surgery can be used to predict late recurrence. Bone marrow samples obtained before and after surgery were analysed for the presence of DTCs using a multimarker mRNA quantitative reverse transcription PCR assay, alongside other established markers such as the mitotic activity index (MAI) and lymph node status. Survival analysis showed that patients with pre-operative DTCs recurred much earlier compared to the DTC-negative patients. A high MAI count was also associated with shorter overall survival. Univariable and multivariable Cox regression analyses showed that the highest risk of recurrence was observed in patients who were DTC-positive before and after surgery, with hazard ratios of 6.93 and 8.34, respectively. Furthermore, the presence of DTCs before surgery was the only predictor of late recurrence. This paper supports the idea that DTCs could serve as a biomarker to identify patients at risk of recurrence due to cancer dormancy who may benefit from extended therapy or more frequent monitoring by healthcare professionals.

While transcriptomic signatures can, in principle, be used as clinical tools, as demonstrated by clinical assays such as Oncotype DX in breast cancer [89], Tian et al.’s dormancy-associated signature remains preliminary, as it is derived from a retrospective cohort using a literature-derived gene panel for cancer dormancy. Given the lack of a clear molecular definition for cancer dormancy in the literature, without prospective validation and a standardised definition for the form of cancer cell dormancy being predicted, the signature remains correlative rather than predictive. However, Tian et al.’s results represent a step towards predicting cancer dormancy and recurrence. Tjensvoll et al.’s more direct approach, utilising the physical presence of DTCs in bone marrow, also represents a promising direction for the prediction of recurrence as a result of cancer cell dormancy; however, the invasive bone marrow aspiration needed to check for DTCs is invasive and impractical for routine screening, limiting its scalability.

Taken together, these studies demonstrate two distinct computational strategies for understanding how cancer dormancy may influence patient outcomes: mathematical modelling of reactivation dynamics and empirical associations between dormancy features and clinical outcomes. It is worth noting that both strategies remain largely nascent: mathematical models rely on strong simplifying assumptions about dormancy, while association studies are limited by their retrospective, correlative designs. Currently, these strategies operate in parallel: the mathematical models are not parameterised by the molecular data used in association studies, and the association studies do not incorporate the dynamic frameworks provided by the models. Furthermore, currently, neither strategy accounts for the biological complexity established earlier in Section 2. As this field develops, the mechanistic insights discussed in previous sections (e.g., transcriptional programmes, microenvironmental interactions, and epigenetic modifications) will need to be incorporated into these predictive models to improve these computational strategies.

## 3. Current Challenges and Future Perspectives: Computational Analysis of Cancer Dormancy

**Figure 3:**
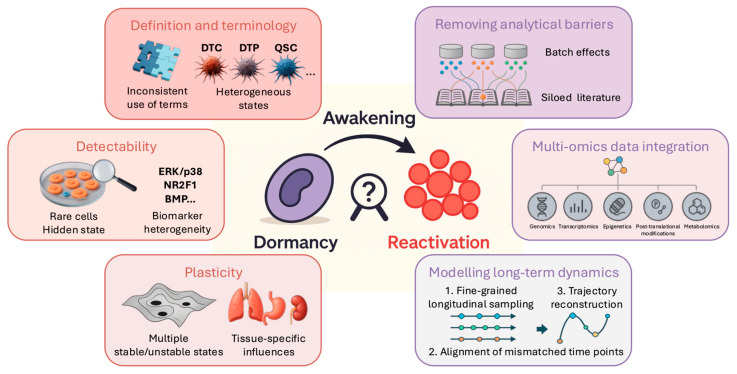
Computational challenges and future directions in modelling cancer cell dormancy. Key analytical barriers (left panels) and proposed solutions (right panels) for advancing computational studies of cancer dormancy. Current limitations include fragmented and inconsistent terminology (e.g., DTC, DTP, QSC - quiescent stem cells), difficulty in detecting rare states, the absence of universal biomarkers, and plasticity in dormancy-state switching, also influenced by the tissue context. Moving forward, improving the characterisation and understanding of this complex process will require addressing analytical barriers such as batch effect and multi-study integration, multi-omic profiling (genomics, transcriptomics, epigenomics, proteomics, metabolomics) of dormant cells, and fine-grained longitudinal sampling combined with dynamic modelling frameworks to reconstruct entry, maintenance, and reactivation trajectories. The visual elements in this figure have been generated with the help of Microsoft Copilot version bizchat.20260416.29.2.

In Section 2, we reviewed the main types of computational inference used to investigate cancer dormancy. This revealed significant methodological diversity but also highlighted key recurring limitations, including reliance on scRNA-seq, fragmented definitions of cancer dormancy, and difficulty combining datasets for meta-analysis. It is also worth noting that the studies in Section 2 are dominated by a small number of cancer types, with breast cancer in particular dominating the scRNA-seq literature. Cancer dormancy remains computationally underexplored in other cancer types, so it is not clear whether the molecular programmes and analytical frameworks discussed in Section 2 apply to the lesser-studied cancer types. In this section, we begin by discussing the main challenges in computational cancer dormancy research, then outline proposed solutions and future directions for the field. Figure 3 provides a schematic overview linking each challenge to corresponding computational strategies.

### 3.1. The Ground Truth Problem

Despite extensive work characterising dormant cancer cells, there is still no agreed-upon gold standard for computationally identifying cancer dormancy [90,91]. This likely reflects the sheer diversity of cell types encompassed by the term “dormant cancer cells.” Although all forms of cancer dormancy share longer-lived and reversible G0/G1 cell cycle arrest, the mechanisms governing entry into, maintenance of, and exit from dormancy are not necessarily shared [92]. A range of factors, including cancer type, prior chemotherapy exposure, the specific agents used, and even the niche in which the cells reside, are likely to influence how dormancy is regulated. Therefore, it is not surprising that the field has struggled to identify a universal marker of cancer dormancy; even well-established subtypes such as DTCs and DTPs lack well-defined markers [29,93]. Despite their growing clinical relevance, PGCCs remain computationally understudied compared to DTPs and DTCs, with the few existing examples limited to standard differential expression and pathway enrichment [94]. Extending more advanced computational approaches to other emerging forms of dormancy remains an important future direction.

This issue is further amplified by inconsistent methodological approaches across studies, in which different studies may apply distinct “dormancy signatures” or stratification strategies for dormant cells, hindering the alignment of findings. Consequently, the field may be collectively examining overlapping but not identical dormancy-associated cell states rather than a single discrete identity. Thus, we believe that the lack of consensus markers for cancer dormancy as an umbrella term and even its many proposed subtypes represents one of the largest challenges facing those investigating dormancy computationally.

The absence of standardised marker definitions makes dormancy particularly susceptible to propagation of inconsistent signatures across computational workflows. The lack of consensus markers also becomes apparent when attempting automated literature mining. Computational approaches that can systematically aggregate and reconcile heterogeneous literature may therefore provide a complementary strategy to address this fragmentation. To examine whether recent AI-assisted tools could help mitigate fragmentation in biomarker identification, we compared dormancy-associated genes curated manually by two independent reviewers with those identified by an AI agent using structured prompts. To address this challenge, we developed LitGraph, an AI-assisted literature mining framework designed to systematically extract and structure dormancy-associated knowledge from the literature. LitGraph analyses papers using the Claude large language model and builds knowledge graphs summarising these findings via GraphRAG (see Methods). Conceptually, the LitGraph pipeline operates in three stages: (i) targeted retrieval of domain-specific literature using structured queries, (ii) context-aware extraction of candidate biomarkers and functional roles, and (iii) integration of extracted entities into a graph-based framework that enables relationship tracing across studies. This design allows the system to move beyond isolated gene lists toward a network-level representation of dormancy biology.

Unlike conventional literature mining approaches based on keyword co-occurrence, frequency analysis, or static ontologies, LitGraph is designed to capture structured biological relationships (e.g., gene–function–context triplets) and preserve their contextual meaning across studies. In the context of cancer dormancy, where biomarker definitions are highly fragmented and context-dependent, this enables systematic comparison of reported regulators and helps distinguish consistently supported mechanisms from context-specific or weakly supported claims.

In practice, applying LitGraph to the problem of fragmented dormancy biomarkers allowed us to recover a core set of recurrent regulators, including NR2F1, p38 MAPK, TGFβ2, BMP7, and canonical CDK inhibitors, suggesting that highly cited core mechanisms are consistently retrievable (Tables S1–S5). At the same time, areas of conceptual ambiguity became apparent, where qualitative differences in interpretation were evident. The AI agent frequently conflated experimental readouts used to identify dormant cells (e.g., proliferation or label-retention markers) with mechanistic regulators of dormancy, misattributed claims to incorrect references, or cited papers whose conclusions did not fully support the statement made. Notably, the agent did not generate fictitious citations; rather, inaccuracies arose primarily from contextual misinterpretation or imprecise attribution. Following manual curation, approximately 75% of AI-suggested entries corresponded to real papers from which relevant information could be appropriately extracted, but systematic validation was required.

In practice, although AI-assisted curation accelerated the initial aggregation of candidate markers and occasionally surfaced studies not captured in the first manual pass, the time required for verification reduced the overall efficiency gain. This exercise underscores the broader challenge facing the field: without standardised definitions and clearly annotated reference datasets, both human and automated approaches risk propagating ambiguity. At present, AI language models are best viewed as complementary hypothesis-generation systems rather than authoritative sources. A hybrid workflow, with initial manual grounding followed by AI-assisted expansion and expert validation, may therefore offer a pragmatic interim solution, while the development of a community-maintained, continuously updated reference database of experimentally supported dormancy markers would provide a more durable foundation for computational analyses.

### 3.2. Barriers to Meta-Analysis

Another factor complicating the computational analysis of dormancy is the difficulty in comparing results and/or combining multiple datasets for meta-analysis. Even within a single data modality like scRNA-seq, cross-study comparison and analysis are limited by batch effects, differences in preprocessing, heterogeneous experimental conditions, and a lack of a standardised definition of what counts as a dormant cancer cell. These issues are exacerbated when attempting to compare studies with more complex designs. For example, it is very difficult to compare or integrate results from longitudinal studies because most longitudinal studies sample at different time points and use different treatment schedules. The result is that the majority of the existing dormancy literature is essentially siloed, so individual studies generate their own clusters, signatures, or models, making it very difficult to validate between studies or combine data to generate a larger picture. Despite these barriers, some studies have attempted cross-dataset integration by stepping up to a level of abstraction to the network scale. For example, Uzuner et al. [95] aggregated 21 dormancy-cancer comparisons from 10 bulk transcriptomic datasets across seven cancer types and then mapped the differentially expressed genes onto protein–protein interaction and gene-regulatory networks to extract modules enriched in dormancy-associated genes. This approach is appealing because it somewhat sidesteps the integration problem. Instead of forcing heterogeneous datasets together for analysis, this approach asks which interactions are recurrently implicated across studies. While a promising direction, it is important to note that this approach inherits significant limitations from its inputs: it operates on bulk rather than single-cell data, depends on the completeness of existing interactome resources, and still relies on study-specific definitions of dormant and proliferative states. Network-level meta-analysis therefore offers a useful complementary lens rather than a full solution, and extending this strategy to single-cell and multi-omic datasets remains an open challenge. Beyond practical limitations, the conceptual representation of cancer dormancy also complicates computational analysis.

### 3.3. Cancer Dormancy as a Continuum or a Set of Discrete States

There is emerging evidence that rather than falling into discrete, well-defined categories, dormant cancer cells may occupy a spectrum of arrest depth, ranging from shallow, easily reversible quiescence through to deeper senescence-like states [29,96]. Under this model, subtypes such as DTPs and DTCs could represent overlapping ranges along this quiescence-depth axis. However, other subtypes are defined by features that cannot be reduced to arrest depth alone. PGCCs, for example, are characterised by polyploidy and multinucleation, properties that distinguish them morphologically and mechanistically from other dormant subtypes. This suggests that dormancy heterogeneity may operate across multiple axes simultaneously: a continuum of arrest depth within some subtypes, but discrete morphological and gene regulation distinctions between others. In support of a continuum within dormancy subtypes, Fernandez et al.’s pseudotime analysis (discussed in Section 2.3) showed that drug-induced arrest formed a continuous trajectory from shallow quiescence to deeper senescence-like states rather than discrete transcriptional clusters, though this was observed in non-cancerous MCF10A cells. Whether this within-subtype continuity extends to cancer cells and whether different dormancy subtypes (e.g., DTPs vs. DTCs vs. PGCCs) represent discrete categories or points on a broader shared spectrum remain open questions. The answer to this question has significant consequences for how we study cancer cell dormancy computationally.

If cancer cells can progressively deepen their arrest along a quiescence-to-senescence axis, then clustering-based analyses, commonly used to computationally distinguish dormant cancer cells from proliferative cancer cells, impose artificial boundaries on what may actually be a continuous landscape. Furthermore, if we assume that cancer dormancy is a spectrum, a cell occupying an intermediate state may be mistakenly grouped with fully dormant or fully proliferative cells, thereby clouding downstream biological interpretations. Thus, when studying cancer cell dormancy, researchers must consider how their assumptions about the taxonomic structure of dormant cancer cells affect their chosen analytical techniques. If one assumes discrete cell states, then standard clustering with classifier-based approaches is appropriate, as they assign cells to defined categories with clear boundaries. If one assumes a continuum of cell states, then pseudotime [97] and optimal transport [98] approaches are more suitable, as they preserve continuous ordering and do not impose artificial boundaries on cell states. If the reality is some sort of intermediate between the two, then neither technique alone is sufficient. For example, researchers could apply both clustering and trajectory-based approaches to the same dataset and evaluate which better captures the underlying structure, or apply pseudotime analysis within computationally defined subpopulations to test for continuous variation within otherwise discrete groupings.

Ultimately, more research is needed to understand the landscape of cancer dormancy, particularly to determine whether current categorical frameworks are sufficient or whether novel analytical approaches are required to represent this complexity faithfully.

### 3.4. Beyond Transcriptomics: The Need for Multi-Omic Integration and Modelling

The majority of computational studies interrogating cancer dormancy have relied on scRNA-seq as the primary modality; however, it is increasingly clear that cancer dormancy’s biology likely spans multiple molecular layers that cannot be captured by scRNA-seq alone. For example, the balance between ERK/p38 phosphorylation has been shown to be a hallmark of dormant cancer cells, but these post-translational changes are invisible to scRNA-seq [51,52,53,54,55,56]. Epigenetic changes are another example, with multiple studies described in Section 2 showing that epigenetic changes are involved in cancer dormancy [71,83]. Given that cancer dormancy likely operates across multiple molecular scales, we suggest that, moving forward, multi-omic studies combining multiple modalities at single-cell resolution are needed, alongside appropriate computational integration methods. Methods already exist for combining and analysing different single-cell modalities, for example, Multi-Omic Factor Analysis v2 (MOFA+) [99] or graph-linked unified embedding [100].

### 3.5. Foundation Models and Transfer Learning for Learning Rare Cell States

As previously discussed, identifying dormant cancer cells remains a major obstacle to computational research on cancer dormancy. Furthermore, dormant cancer cells are rare in most snapshot studies, making it difficult to develop supervised classifiers, as models will either overfit to dataset-specific artefacts or collapse cancer dormancy into broader quiescent cell states. Foundation models pre-trained on large amounts of scRNA-seq data could help address this limitation. The general-purpose representations learned by pre-trained foundation models can serve as a base for fine-tuning with relatively few annotated examples of dormant cancer cells. A few such models now exist, for example, Geneformer, a foundational model pre-trained on approximately 30 million scRNA transcriptomes [101]; scGPT, a foundational model that is based on a generative pretrained transformer on approximately 33 million scRNA transcriptomes [102]; scFoundation, a foundation model pre-trained on 50 million human scRNA-seq profiles [103]; or scBERT, a pre-trained deep neural network-based model focused on cell type annotation [104]. Importantly, the biggest challenge here will not be identifying dormancy but delineating normal dormancy from cancer dormancy, which may require signatures from different modalities, such as epigenetic or metabolic signatures that current foundation models do not capture.

### 3.6. Temporal Dynamics: Continuous-Time Models

The majority of the computational analyses described in Section 2 were applied to snapshot data, but cancer dormancy is defined by temporal behaviour (entry, dwell time, and exit). For example, a cancer cell that may appear transcriptionally quiescent in scRNA-seq data from a single moment in time could be transiently pausing before re-entering the cell cycle to proliferate again or remain stably dormant for decades. To this end, longitudinal studies examining dormancy over time, which have been greatly enabled through lineage tracing or similar experiments, are crucial for understanding cancer dormancy. Integrating multiple longitudinal studies would, in principle, allow us to capture a broader view of the temporal aspects of dormancy; however, as previously explained, it is difficult to integrate multiple longitudinal studies due to mismatched sampling time points. A potential solution to this problem is to use continuous-time models to sidestep the mismatched sampling time issue. For example, a latent ODE framework, as proposed by Rubanova et al., which learns a shared dynamical model defined by a neural ODE that can be evaluated at any time point, irrespective of when the sample was actually collected [105]. In principle, this would allow one to merge data from longitudinal studies with mismatched time points to inform a single model of cancer cell dormancy dynamics. However, it is important to note that there are some fundamental challenges. For example, because scRNA-seq is destructive, individual cell trajectories cannot be observed directly. Therefore, to apply a latent ODE here, one would need to either use population-level summaries (e.g., collapsing scRNA-seq data into an aggregate representation for each time point) at each time point or utilise lineage tracing to link related cells across time points. Another drawback to consider is that longitudinal datasets typically sample only a few time points, which may not be sufficient for learning complex non-linear dynamics. One would likely need to integrate multiple studies and impose strong biological priors.

Taken together, the challenges outlined in this section highlight a central disconnect in computational cancer dormancy research: the biological phenomenon is dynamic, heterogeneous, and multi-layered, whereas many current analytical frameworks remain static, transcriptome-centric, and dataset-specific. Overcoming these limitations will require not only technical innovation but also greater conceptual alignment regarding how dormancy is defined and modelled across studies. As datasets become increasingly longitudinal, spatially resolved, and multi-modal, the opportunity arises to move beyond isolated signatures toward integrated representations of dormancy as a dynamic systems process. Establishing such frameworks will be critical for ensuring that computational advances translate into biologically coherent and clinically actionable insights (Figure 3).

While this review has, for the most part, focused on computational approaches to investigate cancer cell dormancy, it is important to note that these computational approaches are not possible without rigorous experimental research, and the most effective experimental and computational methods are used iteratively. Several of the studies discussed in Section 2 demonstrate this synergy. For example, Khoo et al. used scRNA-seq to identify *AXL* as a marker of dormant cancer cells in myeloma and then validated these results experimentally with coculture and intravital imaging in vivo. Similarly, Hu et al. used computational analyses to interpret results from a CRISPR screen and then validated those analyses pharmacologically, ultimately showing that STING activation reduced the micrometastatic burden. These examples demonstrate just how effective the closed loop is; computational analyses allow us to generate testable hypotheses, whose results can then be used to further refine computational analyses.

A clinically relevant extension of this iterative cycle is the translation of computationally identified dormancy regulators into therapeutic targets. The studies reviewed in Section 2 collectively identified several examples of candidate therapeutic targets. Alongside ongoing experimental efforts to translate such candidates into cancer treatments [106,107,108], computational techniques such as network-based drug target prioritisation [109], drug sensitivity prediction [110,111], and drug repurposing approaches [112] could, in the future, help bridge the gap between molecular insights derived from computational studies and cancer therapies.

## 4. Materials and Methods

To investigate fragmentation in cancer dormancy biomarker identification, we compared manual literature curation with an automated AI-assisted pipeline developed in-house (LitGraph). The objective was to evaluate concordance, divergence, and systematic error patterns between human and AI-driven biomarker extraction workflows. The study consisted of three components:

• Manual biomarker curation by two independent reviewers.
• AI-assisted literature mining using the LitGraph framework.
• Manual reviewing and structured comparison of outputs by a third reviewer.

### 4.1. Manual Literature Curation 

Two independent reviewers manually curated molecular regulators associated with cancer cell dormancy/quiescence from peer-reviewed literature. Inclusion criteria required experimental evidence supporting a mechanistic role in entry into, maintenance of, or exit from cellular dormancy. Experimental readouts used solely to identify dormant cells (e.g., label-retention dyes or proliferation markers such as Ki67) were distinguished from mechanistic regulators.

### 4.2. AI-Assisted Literature Mining Using LitGraph

AI-assisted curation was performed using LitGraph, an automated literature analysis pipeline developed by the authors and publicly available at https://github.com/RunningStone/LitGraph (accessed on 26 September 2025).

LitGraph is designed to automatically query literature databases, retrieve and process publications, analyse textual content using a large language model, and construct structured knowledge graphs summarising extracted relationships.

In this study, LitGraph was configured to retrieve literature related to cancer dormancy and quiescence published between 1995 and 2024 using predefined keyword queries. Retrieved documents were processed and analysed using the Claude large language model (Anthropic) for structured extraction of:

• Candidate biomarkers
• Claimed functional roles
• Associated references
• Supporting evidence statements

Extracted entities and relationships were organised using a GraphRAG (Graph-based Retrieval-Augmented Generation) framework. This enabled:

• Construction of a structured knowledge graph linking biomarkers to mechanisms and references
• Relationship tracing between signalling pathways, transcription factors, and dormancy states
• Context-aware retrieval of supporting claims.

The AI-generated biomarker tables (Tables S1–S3) were exported in a structured format to enable direct comparison with the manually curated dataset (Table S4), and a final synthesis table was generated (Table S5).

### 4.3. Verification and Error Classification

All AI-derived entries were manually verified against the cited primary literature. Each entry was classified into one of the following categories:

• Correct citation and accurate contextual extraction
• Real paper cited with incorrect bibliographic details
• Real paper cited but claim not supported by source
• Conceptual conflation (e.g., experimental identification marker misclassified as mechanistic regulator)
• Non-verifiable claim

No fictitious (non-existent) publications were identified during review. Approximately 75% of AI-suggested entries corresponded to real papers from which relevant dormancy-related information could be extracted following adjudication. An annotated comparison table detailing validation outcomes is provided in Table S5.

## 5. Conclusions

Cancer dormancy reflects a form of cell state plasticity with profound clinical consequences. Although increasingly accessible through modern profiling technologies, it remains resistant to simple classification, suggesting that dormancy is better understood as a dynamic and context-dependent process rather than a fixed cellular identity. Computational approaches have begun to reshape how dormancy is conceptualised: not merely as a rare quiescent population, but as a systems-level phenomenon governed by state transitions across molecular and temporal scales. The challenge ahead is to translate these conceptual advances into coherent modelling frameworks and clinically interpretable predictors. Achieving this will be essential if dormancy biology is to move beyond descriptive characterisation toward strategies that anticipate and prevent cancer recurrence.

## Data Availability

Curated biomarkers with contextual synthesis, literature mappings, and annotated comparisons are provided in Table S1. No new datasets were generated. All referenced data originate from previously published studies cited in the manuscript. The LitGraph framework used for biomarker retrieval in this study is publicly available at the following repository: https://github.com/RunningStone/LitGraph (accessed on 26 September 2025).

## Supplementary Materials

The following supporting information can be downloaded at https://www.mdpi.com/article/10.3390/biom16050633/s1. Table S1: Structured biomarker annotation table derived by LitGraph from curated literature and contextual validation. Table S2: Contextual interpretation table for expert quality control and semantic validation. Table S3: Literature cross-reference table linking extracted entries to original publications. Table S4: Cancer dormancy biomarkers retrieved by human experts through manual curation of the literature. Table S5: Comparative evaluation of manually and AI-curated cancer dormancy biomarkers.

## Abbreviations

The following abbreviations are used in this manuscript:

AI	Artificial Intelligence
AMPK	AMP-Activated Protein Kinase
CDK	Cyclin-Dependent Kinase
ChIP-seq	Chromatin Immunoprecipitation Sequencing
CRISPR	Clustered Regularly Interspaced Short Palindromic Repeats
CTC	Circulating Tumour Cell
DTC	Disseminated Tumour Cell
DTP	Drug-Tolerant Persister
ECM	Extracellular Matrix
ERK	Extracellular Signal-Regulated Kinase
GSEA	Gene Set Enrichment Analysis
GraphRAG	Graph-based Retrieval-Augmented Generation
IIS	Insulin/Insulin-like Growth Factor Signalling
LC-MS/MS	Liquid Chromatography–Tandem Mass Spectrometry
MOFA	Multi-Omic Factor Analysis
NMF	Non-negative Matrix Factorisation
NRF2	Nuclear Factor Erythroid 2–Related Factor 2
ODE	Ordinary Differential Equation
PGCC	Polyploid Giant Cancer Cell
scRNA-seq	Single-Cell RNA Sequencing
sgRNA	Single Guide RNA
SRCC	Stably Resistant Cancer Cell
super-SILAC histone mass spectrometry	Super-Stable-isotope labelling by amino acids in cell culture histone mass spectrometry
TOR	Target of Rapamycin
